# Clinical Features and Outcomes of Primary Sclerosing Cholangitis in the Highly Admixed Brazilian Population

**DOI:** 10.1155/2021/7746401

**Published:** 2021-11-10

**Authors:** Mateus Jorge Nardelli, Paulo Lisboa Bittencourt, Guilherme Grossi Lopes Cançado, Luciana Costa Faria, Cristiane Alves Villela-Nogueira, Vivian Rotman, Eliabe Silva de Abreu, Fernanda Maria Farage Osório, Andreia Silva Evangelista, Liliana Sampaio Costa Mendes, Daniel Ferraz de Campos Mazo, Elodie Bonfim Hyppolito, Adrielly de Souza Martins, Liana Codes, Izabelle Venturini Signorelli, Geisa Perez Medina Gomide, Luciana Agoglia, Claudia Alexandra Pontes Ivantes, Valéria Ferreira de Almeida e Borges, Gabriela Perdomo Coral, Rosamar Eulira Fontes Rezende, Maria Lucia Gomes Ferraz, Debora Raquel Benedita Terrabuio, Eduardo Luiz Rachid Cançado, Claudia Alves Couto

**Affiliations:** ^1^Hospital das Clínicas, Universidade Federal de Minas Gerais, Belo Horizonte, Brazil; ^2^Hospital Português, Salvador, Brazil; ^3^Escola Bahiana de Medicina e Saúde Pública, Salvador, Brazil; ^4^Hospital da Polícia Militar de Minas Gerais, Belo Horizonte, Brazil; ^5^Faculdade de Medicina, Hospital Universitário Clementino Fraga Filho, Universidade Federal do Rio de Janeiro, Rio de Janeiro, Brazil; ^6^Hospital de Base do Distrito Federal, Brasília, Brazil; ^7^Faculdade de Ciências Médicas, Universidade Estadual de Campinas, Campinas, Brazil; ^8^Hospital Universitário Walter Cantídio, Universidade Federal do Ceará, Fortaleza, Brazil; ^9^Hospital Universitário Professor Edgard Santos, Salvador, Brazil; ^10^Hospital Universitário Cassiano Antônio Moraes, Vitória, Brazil; ^11^Hospital das Clínicas da Universidade Federal do Triângulo Mineiro, Uberaba, Brazil; ^12^Hospital Universitário Antônio Pedro, Universidade Federal Fluminense, Niterói, Brazil; ^13^Hospital Nossa Senhora das Graças, Curitiba, Brazil; ^14^Universidade Federal de Uberlândia, Uberlândia, Brazil; ^15^Irmandade da Santa Casa de Misericórdia de Porto Alegre, Porto Alegre, Brazil; ^16^Hospital das Clínicas da Faculdade de Medicina de Ribeirão Preto, Universidade de São Paulo, Ribeirão Preto, Brazil; ^17^Universidade Federal de São Paulo, São Paulo, Brazil; ^18^Faculdade de Medicina da Universidade de São Paulo, São Paulo, Brazil

## Abstract

**Background:**

Primary sclerosing cholangitis (PSC) is associated with a broad phenotypic spectrum in different populations from diverse ethnic and racial backgrounds. This study aimed to describe the clinical characteristics and outcomes of PSC in a multicenter cohort of patients from Brazil.

**Methods:**

Data from the Brazilian Cholestasis Study Group were retrospectively reviewed to assess demographic information and clinical characteristics of PSC, as well as the outcomes, such as transplantation-free survival.

**Results:**

This cohort included 210 patients. After excluding 33 (15.7%) patients with PSC and overlap syndrome of autoimmune hepatitis, 177 (97 males, median age 33 (21–42) years) with clear-cut PSC were eligible for this study. Most of the patients (*n* = 139, 78.5%) were symptomatic, and 104 (58.7%) had advanced PSC at the time of diagnosis. Concurrent inflammatory bowel disease was observed in 78 (58.6%) of the investigated patients (*n* = 133), and most of them had ulcerative colitis (*n* = 61, 78.2%). The 1- and 5-year survival free of liver transplantation or death were 92.3 ± 2.1% and 66.9 ± 4.2%, respectively, and baseline advanced PSC, pruritus, and elevated bilirubin levels were independent risk factors for the composite adverse outcome. Females were significantly older and had lower bilirubin levels than males at baseline, but survival was not associated with sex. Approximately 12.4% (*n* = 22) of patients with PSC died, and 32.8% (*n* = 58) underwent liver transplantation at a median follow-up time of 5.3 and 3.2 years.

**Conclusion:**

Multiethnic Brazilian PSC patients exhibited a less pronounced male predominance and a lower frequency of inflammatory bowel disease than Caucasians. Adverse outcomes were more frequent, probably due to advanced disease at baseline.

## 1. Introduction

Primary sclerosing cholangitis (PSC) is a rare chronic cholestatic liver disease of unknown etiology with a wide spectrum of clinical features [1]. Although studies in northern Europe and the United States have extensively characterized the characteristics of PSC in Caucasians [2–6], there is a paucity of data on disease expression in other parts of the world, particularly in the multiracial and highly admixed population of Latin America [7, 8]. With the emergence of several reports from Asia and western and southern European countries, it has been observed that PSC exhibits a much more varied phenotype and lower disease prevalence than those in other geographical areas [9, 10]. Studies from Spain [11], Singapore [12], Korea [13], and Japan [14, 15] have reported a much lower prevalence of disease in the general population compared to previous northern European studies [16] and clinical characteristics that differ from those classically described, such as a bimodal age of distribution [8, 15] and a lower frequency of concurrent inflammatory bowel disease (IBD) [17–22]. Furthermore, the marked male predominance observed in northern European patients with PSC has not been reported in other populations [15, 17, 18]. However, some studies [1, 5, 23] have suggested that PSC in females may be underdiagnosed as it tends to be more quiescent and less aggressive than PSC in males.

To our knowledge, there are no studies from Latin America that describe the clinical characteristics of patients with PSC [8]. Brazil has a population of highly admixed origin, with varying proportions of genetic ancestry of Native American, African, and European origins, shaped by local historical interactions between migrants brought by the slave trade and European settlement and the Amerindian population [24]. To gather data on the clinical characteristics of PSC and primary biliary cholangitis in the country, the Brazilian Society of Hepatology sponsored a multicenter cooperative consortium named the Brazilian Cholestasis Study Group [25]. This study aimed to describe the clinical characteristics and outcomes of PSC in Brazilians and evaluate the influence of sex on disease expression and outcomes.

## 2. Patients and Methods

### 2.1. Study Population and Case Definition

Patients diagnosed with PSC between 1991 and 2021 in 23 different hepatology centers across the country were enrolled in this retrospective study. The inclusion criteria were the diagnosis of PSC, which was considered in the presence of cholestasis and compatible imaging characteristics of PSC disclosed either by magnetic resonance imaging or endoscopic retrograde cholangiography, as recommended by national and international guidelines [26–28]. Small duct PSC was considered in the presence of typical histological features of PSCs in patients with IBD with normal bile ducts on cholangiography [26, 27]. In this study, the overlap syndrome of autoimmune hepatitis (AIH) and PSC was defined based on typical findings of PSC in patients with additional diagnostic criteria for AIH, as suggested by the International Autoimmune Study Group [29]. The exclusion criteria included the diagnosis of other concomitant liver diseases, including overlap syndrome. Clinical, endoscopic, cross-sectional imaging, and histological data were used to define IBD and its subtypes according to established guidelines [30, 31]. Advanced PSC was considered based on the presence of Ludwig PSC stages III or IV [32] whenever liver biopsy results were available or in the presence of findings compatible with compensated advanced chronic liver disease, such as the presence of esophagogastric varices, irregular external contour of the liver or evidence of collateral circulation on imaging, splenomegaly and low platelet counts [33], or decompensated cirrhosis with variceal bleeding, hepatic encephalopathy, or ascites.

### 2.2. Data Collection

Investigators were asked to identify all patients with PSC who had been followed at their center during the time of the survey and to fill in a standardized database provided by the Brazilian Cholestasis Study Group [25]. Data were retrospectively assessed to evaluate demographic, clinical, and laboratory characteristics of PSC, as well as disease outcomes, such as liver transplantation (LT) and death.

Data collected from medical records included sex, age at diagnosis, baseline clinical and laboratory characteristics, presence of concurrent autoimmune diseases and IBD, treatment with ursodeoxycholic acid (UDCA), outcomes, such as LT or death, and last follow-up visit.

This study was approved by the Federal University of Minas Gerais Ethics Committee Board (CAAE 98626218.6.1001.5149) and conducted following the ethical standards of the Helsinki Declaration.

### 2.3. Statistical Analysis

Statistical analyses were performed using SSPS 25.0 software (IBM, USA). Categorical variables were reported as absolute numbers and percentages. The continuous variable distribution was assessed using the Shapiro–Wilk test, and those with Gaussian distribution were expressed as mean and standard deviation (SD) or as the median and interquartile range (IQR) if the distribution was skewed. Univariate analysis was performed using the chi-square or Fisher's exact test, as appropriate, for categorical variables. Based on the data distribution, continuous variables were analyzed using Student's *t*-test or Mann–Whitney *U* test. Univariate and multivariable Cox regression analyses were used to assess the impact of covariates on combined adverse events (i.e., LT or death). Variables with a *p* < 0.20 were enrolled in the multivariable Cox regression using the backward method, as long as there was no collinearity between variables (i.e., variance inflation factor < 2.5, tolerance > 0.4), and the results were reported as the hazard ratio and 95% confidence interval (95% CI). The Kaplan–Meier method was used to estimate transplantation-free survival, and the log-rank test was performed to compare the survival distributions between the two groups. Statistical significance was set at *p* < 0.05.

## 3. Results

### 3.1. Patient Characteristics

**Figure 1:**
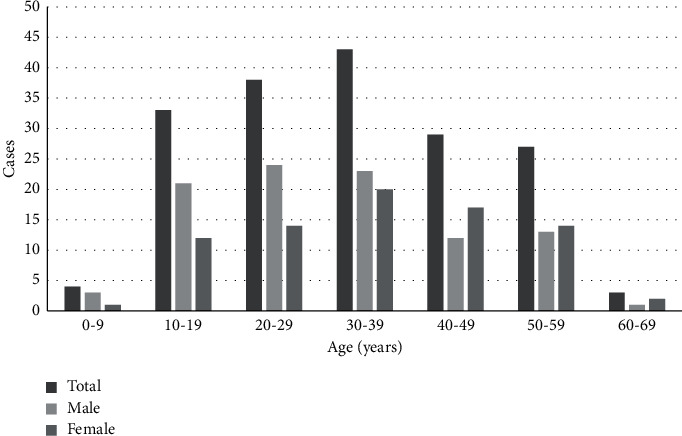
Distribution of patients' age at PSC diagnosis according to sex (*n* = 177).

**Figure 2:**
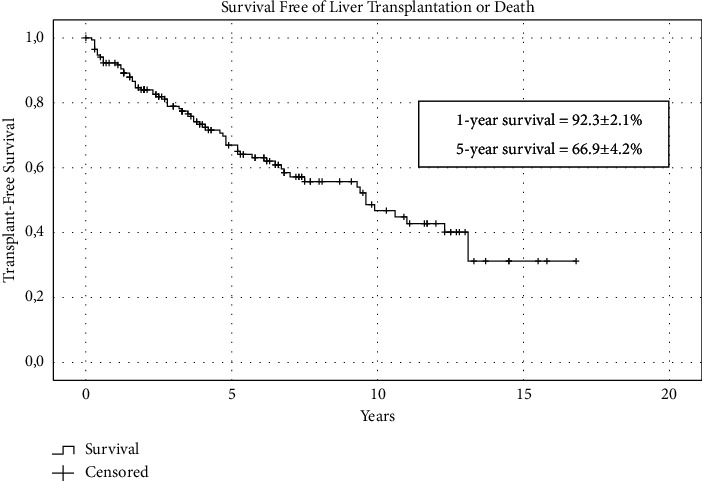
Kaplan–Meier curves of transplant-free survival of patients with PSC (*n* = 177).

The initial cohort included 210 patients with PSC. Thirty-three (15.7%) were diagnosed with PSC and AIH overlap syndrome and, therefore, were excluded. The remaining individuals were eligible for inclusion.  Table 1 summarizes the clinical and laboratory data of the remaining 177 patients with PSC (54.8% males, median age 33 (21–42) years).  Figure 1 shows the age distribution of patients with PSC. The majority of patients were within 20–39 years of age (*n* = 81, 45.8%) and presented symptoms (*n* = 139, 78.5%) at the time of PSC diagnosis, mainly jaundice (*n* = 93, 52.5%) and pruritus (*n* = 78, 44.1%). Supplementary Figure 1 shows the distribution of the study entry decades (i.e., 1991–2001, 2001–2011, and 2011–2021). Most of the patients had large duct PSC (*n* = 154, 87%), and 33 patients were screened for concurrent IBD and 58.6% of them had ulcerative colitis (UC) (*n* = 61), Crohn's disease (CD) (*n* = 13), or indeterminate colitis (*n* = 4). The remaining patients refused to undergo colonoscopy or cross-sectional imaging, mainly due to the absence of symptoms. The mean age at the time of IBD diagnosis was 26 ± 12 years in males and 32 ± 16 years in females (*p*=0.097). The date of IBD diagnosis was available in 67 of 78 cases of IBD. The diagnosis of IBD was performed at a median 1 year before PSC diagnosis (IQR: 6 years before the diagnosis of PSC to 1-year postdiagnosis). IBD was diagnosed before, at the same time, or after PSC diagnosis in 42/67 (62.7%), 17/67 (25.4%), and 8/67 (11.9%) patients, respectively. Advanced PSC was present at baseline in 104 patients (58.7%). Neoplasms were observed in only 11 patients (6.2%). The majority of the patients (*n* = 142, 80.2%) were treated with UDCA. After a median follow-up of 70 (31–126) months, 22 (12.4%) patients died and 58 (32.7%) underwent LT. The follow-up time to LT or death was 39 (15–74) and 64 ± 51 months, respectively. The Kaplan–Meier survival estimate of the entire cohort is shown in Figure 2. The 1- and 5-year survival rates of these patients were 92.3 ± 2.1% and 66.9 ± 4.2%, respectively.

### 3.2. Factors Associated with Sex

Females with PSC had an older age at diagnosis (36 (23–45) vs. 29 (19–40) years in males, *p*=0.046) and lower baseline bilirubin levels (1.2 (0.6––4.2) vs. 2.3 (0.9––7.6) times the upper limit of normal in males, *p*=0.011) compared to their counterparts. No other clinical or laboratory characteristics, including IBD and disease outcomes, were associated with sex, except the fact that females had IBD diagnosis more frequently before (79.3% vs. 50.0%; *p*=0.007) and less frequently after (6.9% vs. 39.5%; *p*=0.007) the detection of PSC than males.

### 3.3. Predictors of Adverse Outcomes

Univariate analysis of clinical and laboratory parameters associated with adverse outcomes (death or LT) showed that symptomatic presentation, pruritus, weight loss, alkaline phosphatase, total bilirubin, and advanced PSC at baseline were associated with adverse outcomes. In the multivariable analysis, pruritus, total bilirubin, and advanced PSC were independently associated with mortality or LT (Table 2). Other variables, including sex and the presence of concurrent IBD or UDCA treatment, were not related to death and/or LT. Supplementary Table 1 provides the 5- and 10-year survival free of death and/or LT for each of the categorical variables analyzed in Table 2.

## 4. Discussion

This study evaluated 177 Brazilian patients diagnosed with PSC. Most patients were aged 20–39 years old, almost half of them were females, and less than 60% had concurrent IBD. The majority of patients had symptoms at presentation and signs of advanced PSC. Females were diagnosed at an older age with lower baseline bilirubin levels. The 1- and 5-year transplantation-free survival rates were 92.3% and 66.9%, respectively, and the outcomes were independently associated with baseline advanced liver disease, pruritus, and elevated bilirubin levels.

To our knowledge, this is the first study from Latin America to describe the demographics, clinical characteristics, and outcomes of patients with PSC [8]. Our findings are divergent from those of previous reports from the United Kingdom [3, 34], the US [2, 35], and Scandinavia [4, 36, 37], which reported a marked male preponderance and a higher frequency of concurrent IBD observed, respectively, in 62–68% and 62–81% of those patients with PSC, but consistent with other studies from western and southern Europe and Asia [15, 22, 38], which have shown a higher frequency of female patients with PSC and a lower prevalence of concurrent IBD. The largest study to date on the phenotype of PSC was conducted by the International PSC Study Group, which evaluated more than 7.000 patients with PSC [5]. In this study, 65.5% of the patients were male, and 70% had concurrent IBD, but most of the patients were recruited from centers in northern Europe, the British Isles, Germany, and North America. Different results were reported by a recent meta-analysis that evaluated the global incidence and prevalence as well as the phenotype of patients with PSC from different parts of the world [8]. The authors have described heterogeneity in incidence and prevalence rates, which were generally much higher in reports from northern Europe and North America than in southern Europe and Asia [8]. In these studies, male predominance and frequency of IBD tended to be less marked in low-prevalence regions than in high-prevalence regions such as northern Europe. The lower frequency of concurrent IBD in this study was aligned with studies from southern Europe and Asia [11, 15, 18]. As reported elsewhere [5, 8], UC was much more common than CD; but different from other reports [5, 39], no association was found between sex and the occurrence of concurrent IBD. Mehta et al. also observed the bimodal age distribution of PSC with relative peaks of age between 15 and 35 years [8]. These findings were previously described in Japan, but different age peaks were observed in the third and seventh decades of life. In this study, no bimodal distribution was observed since most of our patients were in the third to fourth decades of life, as previously described elsewhere [1, 3–5]. However, it is impossible to exclude the impact of referral bias, since all of our patients were recruited from hepatology centers that treat with liver disease in adults.

In this study, females with PSC were diagnosed at an older age compared to males and probably had less advanced disease, considering their baseline levels of bilirubin. A previous study have reported that females were significantly older at the time of disease presentation than males [5] and had a less severe and progressive disease [5]. Based on these findings, it has been hypothesized that the later onset of the disease and the milder course of PSC observed in females may be due to a slower progression of the disease from subclinical disease to full-blown PSC [1, 5, 8, 23]. However, it must be noted that survival in this study was not associated with sex.

Survival in patients with PSC is highly variable due to several factors, including demographics, the occurrence of symptoms, presence of large duct vs. small duct PSC, concurrent IBD and/or cholangiocarcinoma, baseline bilirubin levels, clinical, biochemical, imaging and/or histological signs of advanced disease, presence of portal hypertension, decompensated liver disease, and access to LT [1, 7, 40, 41]. Few studies have been conducted on the natural history of PSC in Latin America, Africa, and Asia [7], and there is still a gap in the knowledge of disease outcomes in underrepresented regions. This study revealed a lower survival of the disease compared to other cohorts of patients [41], particularly in those with advanced PSC, pruritus, and higher baseline bilirubin levels. Compared to other reports [3, 6, 15, 20, 22, 35, 36, 38, 39, 42, 43], a higher percentage of our patients underwent LT due to the presence of advanced disease at baseline or possibly due to referral bias, as some of our patients were enrolled in tertiary care centers with the availability of LT. Taken together, these findings may also highlight that the diagnosis of PSC may be delayed in Brazil or not suspected or screened properly in patients with IBD.

Interestingly, our reported frequency of cholangiocarcinoma was lower than that reported in previous reports [44]. However, the prevalence of cholangiocarcinoma widely varies between different studies, which can be due to the population evaluated (i.e., transplantation centers tend to report a higher number of cases compared to population-based studies) or due to the diagnostic method available in each healthcare facility, as there is a lack of accurate diagnostic modalities to detect early stage cholangiocarcinoma, and surveillance remains controversial between different recommendations [1].

Our study has several limitations, considering its retrospective design and referral bias due to the inclusion of more severe patients from tertiary care centers; however, to our knowledge, this is the first large study addressing PSC characteristics and outcomes in Latin America, with a great contribution not only to local practice but also to the knowledge of PSC expression and outcomes in a multiethnic cohort of patients outside Europe and North America. Although the ethnicity or race of the patients was not reported in this study, it is well described that Brazil has one of the most heterogeneous genetic constitutions in the world, resulting from more than 500 years of interethnic crosses [24]. Furthermore, although our study has assessed factors associated with adverse outcomes in PSC, we did not use previously validated prognostic scores due to their limited usage in clinical practice and lack of validation in the Brazilian population [1, 41], which is characterized for the first time in the literature.

In summary, PSC in Brazilians has a less pronounced male predominance and a lower frequency of concurrent IBD. Females with PSC are diagnosed later in life than males and have a less severe disease at diagnosis, considering baseline bilirubin levels. Survival appeared to be worse, probably due to the more advanced disease at baseline.

## Figures and Tables

**Table 1:** Demographics, clinical, and laboratory features of patients with PSC according to sex.

Variables	All patients (*n* = 177)	Male (*n* = 97)	Female (*n* = 80)	*P* value

Age at diagnosis (years)	33 (21–42)	29 (19–40)	36 (23–45)	**0.046** ^4^
Smoking	18 (10.2)	13 (13.4)	5 (6.2)	0.107^1^
Baseline clinical features				
Asymptomatic	38 (21.5)	19 (19.6)	19 (23.8)	0.502^1^
Jaundice	93 (52.5)	53 (54.6)	40 (50.4)	0.538^1^
Pruritus	78 (44.1)	39 (40.2)	39 (48.8)	0.255^1^
Fatigue	54 (30.5)	29 (29.9)	25 (31.3)	0.846^1^
Weight loss	44 (24.9)	28 (28.9)	16 (20.0)	0.174^1^
Baseline laboratory results (x ULN)				
Aspartate aminotransferase	1.9 (1.2–3.2)	1.8 (1.3–3.3)	2.1 (1.1–3.2)	0.472^4^
Alanine aminotransferase	1.7 (0.9–3.2)	1.7 (1.0–3.2)	1.7 (0.9–3.2)	0.997^4^
Alkaline phosphatase	2.6 (1.6–4.4)	2.7 (1.5–4.4)	2.4 (1.6–4.5)	0.989^4^
Gamma-glutamyl transferase	5.6 (2.8–11.3)	5.6 (2.7–10.7)	5.6 (2.9–12.1)	0.970^4^
Total bilirubin	1.8 (0.7–6.1)	2.3 (0.9–7.6)	1.2 (0.6–4.2)	**0.011** ^**4**^
Serum albumin (mg/dL)	3.8 (3.1–4.3)	3.8 (2.9–4.4)	3.8 (3.3–4.1)	0.897^4^
Platelets count (×10^9^/mm^3^)	205 (120–300)	187 (101–286)	213 (126–314)	0.581^4^
Imaging findings				
Small duct PSC	23 (13.0)	16 (16.5)	7 (8.8)	0.127^1^
Large duct PSC	154 (87.0)	81 (83.5)	73 (91.2)	
IBD investigated	133 (75.1)	73 (75.2)	60 (75.0)	0.968^1^
IBD	78 (58.6)	45 (61.6)	35 (55.0)	0.439^1^
Age at IBD diagnosis (years)	28 ± 14	26 ± 12	32 ± 16	0.097^3^
Ulcerative colitis	61 (78.2)	37 (82.2)	24 (72.7)	0.414^2^
Crohn's disease	13 (16.7)	7 (15.6)	6 (18.2)	
Indeterminate colitis	4 (5.1)	1 (2.2)	3 (9.1)	
Concurrent disorders				
Seronegative rheumatoid arthritis	7 (4.0)	2 (2.1)	5 (6.3)	0.247^2^
Cholelithiasis	29 (16.4)	15 (15.5)	14 (17.5)	0.716^1^
Gallbladder polyps	2 (1.1)	1 (1.0)	1 (1.3)	>0.999^2^
All cancers	11 (6.2)	6 (6.2)	5 (6.3)	>0.999^2^
Colorectal cancer	3 (27.3)	2 (33.3)	1 (20.0)	0.673^2^
Liver and biliary tract	3 (27.3)	1 (16.7)	2 (40.0)	
Others	5 (45.5)	3 (50.0)	2 (40.0)	
UDCA treatment	142 (80.2)	78 (80.4)	64 (80.0)	0.945^1^
Advanced PSC	104 (58.7)	59 (55.7)	38 (53.5)	0.779^1^
Ludwig score III/IV	28/66 (42.4)	18/37 (48.6)	10/29 (34.5)	0.131^2^
Esophagogastric varices	77 (43.5)	47 (48.5)	30 (37.5)	0.143^1^
Splenomegaly	39 (22.0)	22 (22.7)	17 (21.3)	0.819^1^
Low platelet counts	52 (29.4)	29 (29.9)	23 (28.7)	0.868^1^
Variceal bleeding	31 (17.5)	16 (16.5)	15 (18.8)	0.694^1^
Hepatic encephalopathy	26 (14.7)	17 (17.5)	9 (11.3)	0.240^1^
Ascites	54 (30.5)	29 (29.9)	25 (31.3)	0.846^1^
Follow-up time (months)	70 (31–126)	64 (26–115)	76 (40–140)	0.202^4^
Liver transplantation	58 (32.8)	32 (33.0)	26 (32.5)	0.945^1^
Follow-up until transplantation (months)	39 (15–74)	42 (13–76)	33 (16–75)	0.953^4^
Mortality	22 (12.4)	14 (14.4)	8 (10.0)	0.374^1^
Follow-up until death (months)	64 ± 51	54 ± 48	82 ± 58	0.306^3^

Data are expressed as absolute number (percentage), median (interquartile range), or mean ± standard deviation. IBD, inflammatory bowel disease; PSC, primary sclerosing cholangitis; UDCA, ursodeoxycholic acid; ULN, upper limit of normal. ^1^Chi-square test. ^2^Fisher's exact test. ^3^Student's *t*-test. ^4^Mann–Whitney test.

**Table 2:** Factors associated with adverse outcomes, either liver transplantation or death, in patients with PSC.

Variables	Univariate		Multivariable	
	HR (95% CI)	*P* value	HR (95% CI)	*P* value

Female sex	0.82 (0.50–1.33)	0.422		
Age at diagnosis (years)	1.01 (0.99–1.02)	0.399		
Baseline clinical features				
Asymptomatic	0.24 (0.09–0.59)	**0.002**		
Pruritus	2.37 (1.45–3.88)	**0.001**	**1.88 (1.09–3.23)**	**0.023**
Fatigue	1.61 (0.96–2.71)	0.071		
Weight loss	1.95 (1.17–3.25)	**0.011**		
Laboratory at baseline (x ULN)				
Alkaline phosphatase	1.02 (1.00–1.05)	**0.043**		
Total bilirubin	1.10 (1.07–1.14)	**<0.001**	**1.08 (1.05–1.12)**	**<0.001**
Small duct PSC	0.69 (0.31–1.53)	0.369		
IBD	1.39 (0.81–2.39)	0.229		
UDCA treatment	0.70 (0.40–1.23)	0.221		
Advanced PSC	5.89 (2.91–11.93)	**<0.001**	**6.12 (2.73–13.71)**	**<0.001**

Data are expressed as absolute number (percentage) or median (interquartile range). CI, confidence interval; HR, hazard ratio; IBD, inflammatory bowel disease; PSC, primary sclerosing cholangitis; UDCA, ursodeoxycholic acid; ULN, upper limit of normal. Cox regression was performed.

## Data Availability

The data analyzed during this study are available from the corresponding author upon request.

## Supplementary Materials

Supplementary Materials Supplementary Figure 1. Description of study entry decades for patients with primary sclerosing cholangitis. Supplementary Table 1. Five and 10-year survival free of liver transplantation or death in patients with PSC.
