# Supplementary material for: Clinical Features and Outcomes of Primary Sclerosing Cholangitis in the Highly Admixed Brazilian Population
Source: Can J Gastroenterol Hepatol. 2021 Nov 10;2021:7746401. doi: 10.1155/2021/7746401 (PMC8604588; doi:10.1155/2021/7746401)
Supplement: Supplementary Materials — Supplementary Figure 1. Description of study entry decades for patients with primary sclerosing cholangitis. Supplementary Table 1. Five and 10-year survival free of liver transplantation or death in patients with PSC. [file 7746401.f1.docx]

**Supplementary Table 1: Five and 10-year survival free of liver transplantation or death in patients with PSC.**

| **Variables** | **Events (%)** | **Survival**  **(% ± SD)** | | |
| --- | --- | --- | --- | --- |
|  |  | **5-years** | **10-years** | **P-value** |
| **Overall** | 66 | 66.9 ± 4.2 | 46.8 ± 5.4 | – |
| **Sex** |  |  |  |  |
| Female | 28 (42.4) | 73.1 ± 5.5 | 49.9 ± 8.1 | 0.419 |
| Male | 38 (57.6) | 62.8 ± 5.8 | 44.9 ± 7.4 |  |
| **Symptoms at presentation** |  |  |  |  |
| Asymptomatic | 5 (7.6) | 92.7 ± 5.2 | 66.1 ± 14.6 | **0.001** |
| Symptomatic | 61 (92.4) | 60.5 ± 4.8 | 42.1 ± 5.7 |  |
| **Pruritus** |  |  |  |  |
| Yes | 38 (57.6) | 51.3 ± 6.6 | 35.2 ± 7.3 | **<0.001** |
| No | 28 (42.4) | 79.6 ± 4.7 | 56.0 ± 7.7 |  |
| **Fatigue** |  |  |  |  |
| Yes | 22 (33.3) | 56.2 ± 8.5 | 41.5 ± 9.7 | 0.067 |
| No | 44 (66.7) | 70.5 ± 4.6 | 49.4 ± 6.6 |  |
| **Weight Loss** |  |  |  |  |
| Yes | 23 (34.8) | 48.1 ± 8.5 | 32.1 ± 8.9 | **0.009** |
| No | 43 (65.2) | 74.4 ± 4.4 | 52.4 ± 6.6 |  |
| **Small duct PSC** |  |  |  |  |
| Yes | 7 (10.6) | 79.2 ± 9.6 | 56.3 ± 15.8 | 0.363 |
| No | 59 (89.4) | 66.1 ± 4.4 | 46.3 ± 5.8 |  |
| **IBD** |  |  |  |  |
| Yes | 34/56 (60.7) | 57.7 ± 6.7 | 42.8 ± 8.4 | 0.224 |
| No | 22/56 (39.3) | 70.6 ± 6.7 | 42.1 ± 9.3 |  |
| **UDCA treatment** |  |  |  |  |
| Yes | 50 (75.8) | 68.5 ± 4.5 | 48.2 ± 6.1 | 0.216 |
| No | 16 (24.2) | 64.3 ± 8.7 | 44.2 ± 11.5 |  |
| **Advanced PSC** |  |  |  |  |
| Yes | 57 (86.4) | 54.4 ± 5.4 | 28.2 ± 6.2 | **<0.001** |
| No | 9 (13.6) | 88.0 ± 5.0 | 80.5 ± 7.1 |  |

Data are expressed as absolute number (percentage) or percentage (standard deviation). IBD, inflammatory bowel disease; PSC, primary sclerosing cholangitis; SD, standard deviation; UDCA, ursodeoxycholic acid; ULN, upper limit of normal. Kaplan-Meier estimate and log-rank test were performed.
